# Evaluation of a Wildfire Smoke Forecasting System as a Tool for Public Health Protection

**DOI:** 10.1289/ehp.1306768

**Published:** 2013-07-23

**Authors:** Jiayun Yao, Michael Brauer, Sarah B. Henderson

**Affiliations:** 1 School of Population and Public Health, The University of British Columbia, Vancouver, British Columbia, Canada; 2 Environmental Health Services, British Columbia Centre for Disease Control, Vancouver, British Columbia, Canada

## Abstract

Background: Exposure to wildfire smoke has been associated with cardiopulmonary health impacts. Climate change will increase the severity and frequency of smoke events, suggesting a need for enhanced public health protection. Forecasts of smoke exposure can facilitate public health responses.

Objectives: We evaluated the utility of a wildfire smoke forecasting system (BlueSky) for public health protection by comparing its forecasts with observations and assessing their associations with population-level indicators of respiratory health in British Columbia, Canada.

Methods: We compared BlueSky PM_2.5_ forecasts with PM_2.5_ measurements from air quality monitors, and BlueSky smoke plume forecasts with plume tracings from National Oceanic and Atmospheric Administration Hazard Mapping System remote sensing data. Daily counts of the asthma drug salbutamol sulfate dispensations and asthma-related physician visits were aggregated for each geographic local health area (LHA). Daily continuous measures of PM_2.5_ and binary measures of smoke plume presence, either forecasted or observed, were assigned to each LHA. Poisson regression was used to estimate the association between exposure measures and health indicators.

Results: We found modest agreement between forecasts and observations, which was improved during intense fire periods. A 30-μg/m^3^ increase in BlueSky PM_2.5_ was associated with an 8% increase in salbutamol dispensations and a 5% increase in asthma-related physician visits. BlueSky plume coverage was associated with 5% and 6% increases in the two health indicators, respectively. The effects were similar for observed smoke, and generally stronger in very smoky areas.

Conclusions: BlueSky forecasts showed modest agreement with retrospective measures of smoke and were predictive of respiratory health indicators, suggesting they can provide useful information for public health protection.

Citation: Yao J, Brauer M, Henderson SB. 2013. Evaluation of a wildfire smoke forecasting system as a tool for public health protection. Environ Health Perspect 121:1142–1147; http://dx.doi.org/10.1289/ehp.1306768

## Introduction

As the global climate continues to change, more frequent and intense wildfire events and longer wildfire seasons are expected (Flannigan and Van Wagner 1991; Wotton and Flannigan 1993; Wotton et al. 2010). Wildfire smoke can degrade local, regional, and global air quality (Dirksen et al. 2009; Dutkiewicz et al. 2011; Viswanathan et al. 2006). Exposure to wildfire smoke has been associated with adverse cardiopulmonary health effects, with the most consistent associations being found for respiratory outcomes (Dennekamp and Abramson 2011), including dispensations of respiratory reliever medications (Caamano-Isorna 2011; Elliott et al. 2013), physician and emergency department visits (Henderson et al. 2011; Lee et al. 2009; Rappold et al. 2011), and hospital admissions (Delfino et al. 2009; Henderson et al. 2011; Johnston et al. 2007; Morgan et al. 2010; Tham et al. 2009).

Among the different constituents of the complex smoke mixture, PM_2.5_ (particulate matter ≤ 2.5 μm in aerodynamic diameter) has been the most consistently elevated and widely measured exposure metric (Naeher et al. 2007; Sapkota et al. 2005). Tools conventionally used for estimating wildfire smoke exposures include surface PM_2.5_ monitoring and remote sensing products such as the National Oceanic and Atmospheric Administration’s (NOAA) Hazard Mapping System (HMS; U.S. Department of Commerce 2013), which produces hand-drawn smoke plumes by integrating images from multiple satellites (U.S. Department of Commerce 2013). These tools, however, have important limitations. For example, although monitoring networks may accurately reflect ground-level PM_2.5_ concentrations with adequate temporal resolution, they typically do not cover all populated areas affected by fire smoke, and monitors can fail when affected by heavy smoke or actual fire. On the other hand, data from remote sensing products may cover vast geographic areas, but they cannot measure ground-level concentrations, they have different sampling frequencies, and observations can be obscured by clouds. Furthermore, both of these tools provide only retrospective or near–real-time observations. From the perspective of supporting public health responses during wildfire smoke episodes, prospective information is more desirable.

Forecasts have been implemented for many health hazards, including extreme heat (Hajat et al. 2010), pollen (Pasken and Pietrowicz 2005), and ultraviolet radiation (Burrows et al. 1994). An important motivation for using forecasting tools is to provide prospective information for public health actions in order to mitigate the adverse impacts before the hazards actually occur. To support the utility of forecasts for health protection, it is important to know *a*) whether forecasts are accurate and precise compared with reference measurements, and *b*) whether forecasts are associated with population health responses. Most evaluations of forecasting models address only the first question, but for an exposure without a “gold standard” reference measurement, like wildfire smoke, answering the second question is also important. Here we address both questions in an integrated evaluation of the operational BlueSky Western Canada Wildfire Smoke Forecasting Framework (BlueSky; http://www.bcairquality.ca/bluesky/).

**Figure 1:**
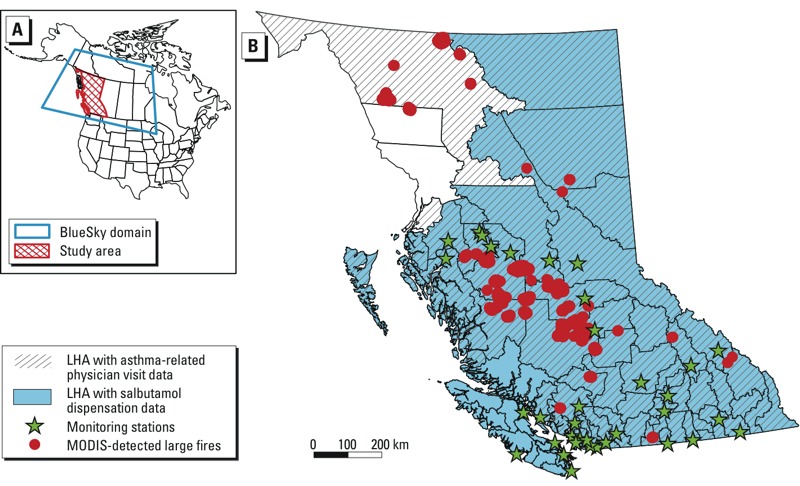
Map of the BlueSky model domain (*A*) and study area (*B*) showing the local health areas (LHAs) and their health data availability, locations of the PM_2.5_ air quality monitoring stations, and locations of fire hot spots detected by the Moderate Resolution Imaging Spectroradiometer (MODIS) with fire radiative power (a measure of fire intensity) > 100 GW. In (*B*) the hatch pattern indicates LHAs with asthma-related physician visit data, and the area in blue indicates LHAs with salbutamol dispensation data.

BlueSky has produced publicly available forecasts of PM_2.5_ concentrations from wildfires up to 60 hr in advance since 2010. Detailed information about the system is described elsewhere (Sakiyama 2013). Briefly, meteorological forecasts, fire locations, fuel consumption estimates, and smoke emissions estimates are combined in a dispersion model to estimate the resulting ground-level PM_2.5_ concentrations in the modeling domain (Figure 1). To date there has been no systematic, quantitative evaluation of general BlueSky performance or of the associations between BlueSky output and population health indicators.

We compared the PM_2.5_ concentrations forecasted by BlueSky with those measured by the ambient air quality monitoring network, and we compared the plume shapes forecasted by BlueSky with those observed by HMS. We then assessed whether respiratory reliever dispensations and asthma-related physician visits show the expected associations with BlueSky forecasts, based on known associations with observed data.

## Methods

*Study area and period*. This study covers the province of British Columbia (BC) in Canada, which is divided into 89 local health areas (LHAs) for administrative purposes (Figure 1). The study period comprised 35 days between 24 July and 29 August 2010 and captured the entire active fire season. Based on area burned, the 2010 fire season was the worst on record in BC. More than 330,000 hectares of forest were burned (Wildfire Management Branch 2013), with the central interior region most severely affected (Figure 1).

*Data description*. BlueSky PM_2.5_. The daily average of PM_2.5_ concentrations was forecasted by BlueSky 48 hr in advance (BlueSky PM_2.5_). Although BlueSky produces PM_2.5_ forecasts up to 60 hr in advance, we present results only for the 48-hr forecasts because this is a relevant averaging period from the public health perspective.

Monitor PM_2.5_. Hourly PM_2.5_ measurements from 36 monitoring stations (32 tapered element oscillating microbalances, 4 beta attenuation monitors) in BC (Monitor PM_2.5_; Figure 1) were retrieved from the BC Ministry of Environment (2012). Midnight-to-midnight 24-hr average concentrations were calculated at each location. The average for any date with six hourly measurements missing in total or three missing consecutively was set to missing. When the filter pressure (a measure of the load on the sampler) was larger than 60%, the sampler was overloaded and the measurement of PM was not reliable, so these data were also set to missing.

BlueSky plumes. Daily smoke plume shapes were derived from the outline of all BlueSky PM_2.5_ forecasting grid cells (0.1° resolution, about 10 km × 10 km; BlueSky Plumes) with daily mean PM_2.5_ values > 0.

HMS plumes. Daily images of smoke plumes from HMS were retrieved from the NOAA HMS website (HMS Plumes; http://www.firedetect.noaa.gov/viewer.html). These plumes were hand-drawn by trained NOAA analysts based on imagery from seven satellites (Ruminski et al. 2006), and each plume was assigned to one of the three semiquantitative smoke density categories. For this study we combined all plumes, regardless of their density categories, observed at different times within a single day to represent areas that had been covered by any HMS plume during any time in that day.

*Population health indicators.* Previous studies have reported significant increases in the asthma drug salbutamol sulfate dispensations (Elliott et al. 2013) and asthma-related physician visits (Henderson et al. 2011) during forest fire smoke episodes in BC. We used similar data to evaluate whether BlueSky output was associated with the same population health indicators. Salbutamol sulfate is commonly used for relief of acute bronchospasm in conditions such as asthma and chronic obstructive pulmonary disease (COPD). Daily counts of the dispensations were extracted from the BC PharmaNet database (BC Ministry of Health 2013) for 85 of the 89 LHAs. Data were not available for four LHAs with populations < 1,000 persons (Figure 1). Outpatient physician visits for asthma were identified as code 493 in the *International Classifications of Diseases, 9th Revision* (World Health Organization 1977). Daily counts were extracted from the BC Medical Services Plan billings database (BC Ministry of Health 2013b) for 73 of the 89 LHAs. Data were not available for 16 LHAs located in the Vancouver Island Health Authority (Figure 1). These two health indicators were divided by the estimated total population of the corresponding LHA in 2010 (BC Stats 2011), resulting in daily rates for each of the LHAs. The overall asthma reliever dispensation and physician visit rates across the province during the study period were 34 and 9.3 per 100,000 person-days, respectively. Both rates decreased during weekends/holidays (18 for salbutamol dispensations and 3.9 for asthma-related physician visits per 100,000 person-days) compared with weekdays (42.7 and 12.3 per 100,000 person-days). A large range of outcome rates was observed across different LHAs, from 17.7 to 72.2 for salbutamol dispensations, and from 2.7 to 21.1 for asthma-related physician visits.

*Exposure assignment*. The health indicator data were aggregated to the LHA level, and we assigned four exposure variables to each LHA for each day of the study period: BlueSky PM_2.5_, Monitor PM_2.5_, BlueSky Plume, and HMS Plume. Because some LHAs cover large geographic areas, we used census dissemination areas (DAs) to estimate population-weighted exposures. One DA typically includes 400–700 people (Statistics Canada 2012), and each LHA contained the geographic centers of multiple DAs, ranging from 3 to 474. We calculated the population-weighted average BlueSky PM_2.5_ and Monitor PM_2.5_ for each LHA using the values at (BlueSky) or nearest to (monitoring stations) the DA centroids. We also overlaid BlueSky Plumes and HMS Plumes with the DA centroids, and LHAs with > 50% of the total population covered by the smoke plumes were assigned a value of 1, and the other LHAs were assigned a value of 0. As a result, two continuous variables (BlueSky PM_2.5_ and Monitor PM_2.5_) and two binary variables (BlueSky Plumes and HMS Plumes) were created.

*Statistical analyses*. Four model evaluation statistics were calculated for the relationship between BlueSky PM_2.5_ and Monitor PM_2.5_: *a*) the Pearson’s correlation coefficient (*r*); *b*) normalized root mean squared error (NRMSE); *c*) index of agreement (IOA, 0–1, where a value of 1 indicates a perfect match); and *d*) fractional bias (FB, difference between observation and forecast divided by the average of the two). These statistics were calculated in three different analyses:

A global analysis, in which all forecasted and measured values at any time and location were included
A spatial-only analysis, in which forecasted and measured values were compared at fixed times for all locations
A temporal-only analysis, in which whole time series of forecasted and measured values were compared for fixed locations.

To quantitatively assess the extent of agreement between BlueSky and HMS plumes, the figure of merit in space (FMS) (Mosca et al. 1998) was calculated. FMS is calculated as the areas of intersection (A_BlueSky_ ∩ A_HMS_, area covered by both BlueSky and HMS plumes) and union (A_BlueSky_ ∪ A_HMS_, area covered by BlueSky and/or HMS plumes) of the two plumes for each day (Equation 1):

FMS = A_BlueSky_ ∩ A_HMS_/ A_BlueSky_ ∪ A_HMS_ × 100%. [1]

We used Poisson regression to estimate the effects of smoke exposure on rates of salbutamol dispensations and asthma-related physician visits. For the continuous variables, the effect was estimated for a 30-μg/m^3^ increase in PM_2.5_, which was equivalent to 2 SDs in the daily PM_2.5_ concentrations measured across all monitoring stations. For the binary variables, the effect of being covered by the smoke plume was compared with not being covered. To account for potential autocorrelation within the time-series data from any individual LHA, parameters in the regression models were calculated with generalized estimation equations (GEEs) in R (R Foundation, Vienna, Austria), assuming an exchangeable correlation structure (where the correlation between all pairs of daily measures within-LHA was uniform and non-zero). Model estimates were adjusted for daily maximum temperature from the closest monitor to each LHA, day of week, holidays, and the week of the study period. A lag of 0–1 days was used for all analyses, based on the best fitted lag time for the acute effects from forest fire smoke in previous BC fire smoke studies (Elliott et al. 2013). The average of the same day and previous day concentrations was used for PM_2.5_, and for the plumes a 1 was assigned if either the same day and/or the previous day had 50% of the population covered.

We also conducted sensitivity analyses to compare areas based on degree of smokiness. Very smoky areas were defined as LHAs covered by HMS smoke plumes for ≥ 12 days (the mean number of days with HMS smoke plumes covering 50% of the population in an LHA), and less smoky areas were defined as other LHAs.

## Results

**Figure 2:**
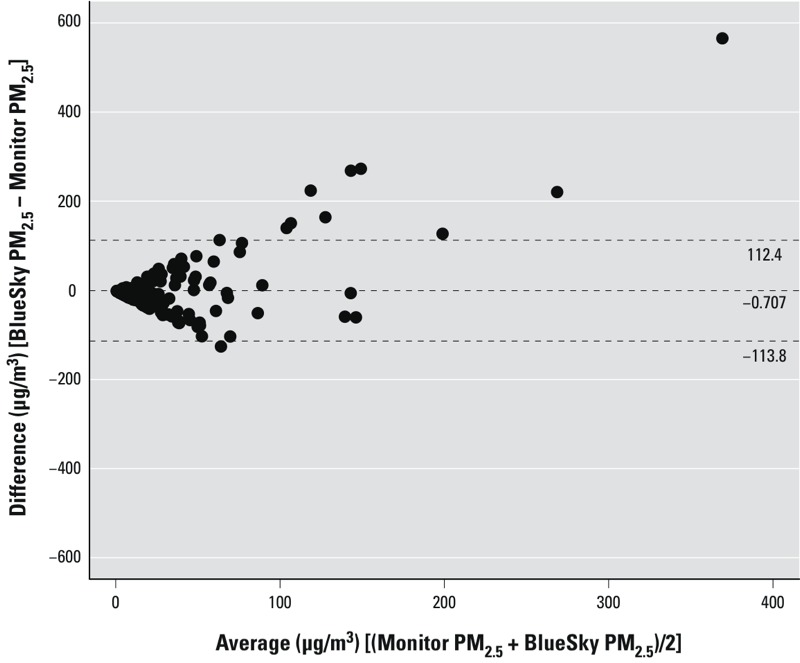
Bland–Altman plot of BlueSky PM_2.5_ versus Monitor PM_2.5_ measurements, excluding all pairs with zero BlueSky predictions. The *x*-axis is the average of BlueSky forecasts and monitor measurements, and the *y*-axis is the difference between the two. Points above zero suggest overpredictions from BlueSky compared with monitors. Dashed lines indicate mean ± 2 SD.

**Figure 3:**
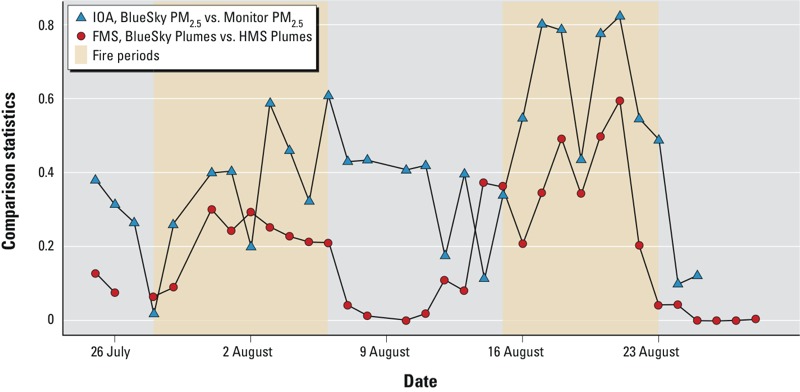
Time-series of daily model evaluation statistics.

*BlueSky PM_2.5_ versus Monitor PM_2.5_*. Model evaluation statistics for BlueSky PM_2.5_ forecasts compared with monitored PM_2.5_ observations showed modest agreement (Table 1). Bland–Altman plots (excluding pairs with zero BlueSky forecasts) indicated that the disagreement between BlueSky PM_2.5_ and Monitor PM_2.5_ was largely attributable to BlueSky overpredictions (Figure 2), also indicated by the negative fractional bias (Table 1). The spatial analyses (Table 1) showed wide ranges in the comparison statistics, suggesting high temporal variability in the spatial agreement, although the time-series plots suggested better agreement during major fire periods (see example of IOA in Figure 3). There was similar variation in comparison statistics for the temporal analyses (Table 1), indicating differing degrees of agreement at different locations. A larger range of NRMSE in the spatial analyses indicated more inconsistent day-to-day spatial agreement than the temporal agreement over all locations.

**Table 1:** Model evaluation statistics in global, spatial, and temporal analyses comparing BlueSky with Monitor PM_2.5_.

Analysis	IOA	*r*	NRMSE (%)	FB
Global	0.53	0.40	18	–0.45
Spatial [mean (range)]	0.41 (0.02, 0.82)	0.32 (–0.17, 0.92)	66 (16, 538)	–0.91 (–2.00, 1.03)
Temporal [mean (range)]	0.46 (0.02, 0.80)	0.31 (–0.36, 0.86)	52 (20, 224)	–1.06 (–1.97, 1.30)
Abbreviations: FB, fractional bias; IOA, index of agreement; NRMSE, normalized root mean squared error; *r*, Pearson’s correlation coefficient.				

The mean Monitor PM_2.5_ ranged from 0.02 to 176.4 μg/m^3^ across the LHAs. The arithmetic mean (± SD) concentration was 10.0 ± 14.5 μg/m^3^, with an interquartile range of 3.0–10.1 μg/m^3^. The mean BlueSky PM_2.5_ forecast ranged from 0 to 988 μg/m^3^ across the LHAs. The arithmetic mean was 4.0 ± 27.3 μg/m^3^ with an interquartile range of 0–0.1 μg/m^3^. The distribution of BlueSky PM_2.5_ was highly skewed to the right because of the large number of zero values in the output. This distribution is typical for air quality model outputs (Mosca et al. 1998), and emphasized for models that only account for one emissions source.

**Figure 4:**
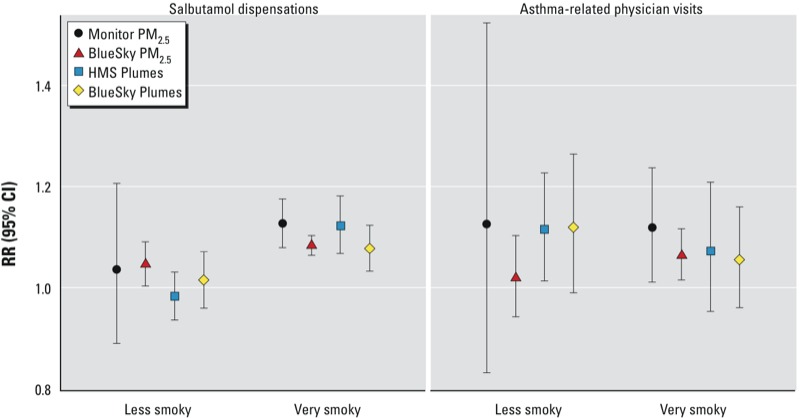
RRs (95% CIs) estimated in very smoky and less smoky areas for salbutamol dispensations (left) and asthma-related physician visits (right).

The rate ratios [RRs (95% CIs)] for salbutamol dispensations were 1.08 (95% CI: 1.06, 1.10) for a 30-μg/m^3^ increase in BlueSky PM_2.5_ and 1.12 (95% CI: 1.07, 1.17) for a 30-μg/m^3^ increase in Monitor PM_2.5_ (Table 2). The RRs for asthma-related physician visits were 1.05 (95% CI: 1.01, 1.09) for BlueSky PM_2.5_ and 1.10 (95% CI: 1.00, 1.21) for Monitor PM_2.5_. Larger point estimates and wider CIs were observed for the Monitor PM_2.5_ compared with the BlueSky PM_2.5_, partially due to some very high concentrations (up to 988 μg/m^3^) forecast by BlueSky. The difference between the two was attenuated when BlueSky PM_2.5_ estimates > 300 μg/m^3^ were truncated to 300 μg/m^3^ (Table 2). In the sensitivity analysis, we found significant associations in very smoky areas, and no associations in less smoky areas, for both health outcome indicators (Figure 4).

**Table 2:** RR (95% CI) for each exposure metric (lag 0–1 in all cases).

Exposure measures	Salbutamol dispensations	Asthma-related physician visits
BlueSky PM_2.5_ (per 30 μg/m^3^)	1.08 (1.06, 1.10)	1.05 (1.01, 1.09)
Truncated BlueSky PM_2.5_ (per 30 μg/m^3^)	1.11 (1.08, 1.13)	1.07 (1.02, 1.12)
Monitor PM_2.5_ (per 30 μg/m^3^)	1.12 (1.07, 1.17)	1.10 (1.00, 1.21)
BlueSky Plumes (1 vs. 0)	1.05 (1.02, 1.09)	1.06 (0.99, 1.15)
HMS Plumes (1 vs. 0)	1.05 (1.01, 1.09)	1.09 (1.02, 1.18)
All models are adjusted for same-day maximum temperature, day-of-week, holiday, and week-of-study. PM_2.5_ values > 300 μg/m^3^ were truncated to 300 μg/m^3^ in truncated BlueSky PM_2.5_.		

*BlueSky Plumes versus HMS Plumes.* The mean areas of BlueSky Plumes and HMS Plumes during the study period were 153,200 and 334,500 km^2^, respectively. BlueSky generally forecasted smaller smoke plumes than those observed by HMS during major fire events. The mean FMS score was 0.21, with a range of 0–0.52. Higher FMS scores were observed during the major fire event periods (Figure 3).

The number of days with ≥ 50% of the population covered by HMS Plumes ranged from 4 to 21 (of 35 days) across the LHAs, with a mean of 12 days. The mean number of days with ≥ 50% of the population covered by BlueSky Plumes was 9 (of 33), with a range of 2 to 21 days. Although the study period was 35 days, 2 days of BlueSky forecasts were missing

The RRs for salbutamol dispensations associated with BlueSky and HMS Plumes were very similar, both with a point estimate of 1.05 (Table 2). The RR for physician visits was 1.06 (95% CI: 0.99, 1.15) for BlueSky Plume coverage and 1.09 (95% CI: 1.02, 1.18) for HMS Plume coverage. In the sensitivity analysis for salbutamol dispensations, we also found significant associations in very smoky areas and no associations in less smoky areas. The same was not observed for the physician visits (Figure 4).

## Discussion

Here we assess a smoke forecasting system for public health protection by *a*) comparing its output with observations from other air quality assessment tools, and *b*) evaluating associations between its output and health indicators known to be associated with those other air quality assessment tools. During short-term air pollution episodes, such as wildfire smoke events, different strategies (ranging from public education to community evacuation) may be implemented based on assessment of exposure levels and their corresponding health risks. BlueSky is one of the many tools available for smoke exposure assessment, but it is different from the other tools because it provides a forecast rather than an observation in near–real-time or a retrospective measure. This study helps to highlight the potential role of smoke forecasting systems in the public health response process.

We found modest agreement between BlueSky PM_2.5_ and Monitor PM_2.5_, with a global correlation of 0.4. The results compared well with correlations of 0.3 and 0.5 between forecasts from the two branches of the European Fire Assimilation System and the MODIS (Moderate Resolution Imaging Spectroradiometer) PM_2.5_ observations (Sofiev et al. 2009). In the comparison of BlueSky Plumes with HMS Plumes, the daily FMS scores ranged from 0 to 0.60, with a mean of 0.18. These scores were slightly higher than the results reported in Stein et al. (2009) for three fire events, where the FMS scores between the U.S. NOAA smoke forecasts and HMS observations ranged from 0.02 to 0.40, with a mean of 0.14. In our study, generally better agreement was observed during intense fire periods, indicated by all evaluation statistics we used.

Disagreement between BlueSky forecasts and observed data could come from the limitations of the BlueSky system, including its inability to predict smoke from fires outside of the modeling domain, and uncertainty in the input meteorology and/or fire information. However, limitations of the HMS plumes and measured PM_2.5_ concentrations are also important because these tools are not gold standards for wildfire smoke exposure assessment or for evaluating BlueSky forecasts. For example, we delineated the shape of BlueSky plume forecasts using all areas where surface concentration estimates were > 0, but the HMS plumes are observed by satellites and therefore reflect smoke in the total column of the atmosphere. On the other hand, air quality monitoring stations capture PM_2.5_ from all sources, whereas BlueSky forecasts only the fraction of PM_2.5_ attributable to smoke from wildfires within the modeling domain (Figure 1). Thus, monitor observations might reflect a large fraction of PM from other sources in areas with limited smoke, affecting the agreement with BlueSky.

We found 8% and 12% increases in salbutamol dispensations associated with 30-μg/m^3^ increases in BlueSky and Monitor PM_2.5_, respectively. The same increases in BlueSky and Monitor PM_2.5_ were also associated with 5% and 10% increases in asthma-related physician visits. The RRs for BlueSky were smaller than, but comparable with, monitor observations in this study and other similar studies. Elliott et al. (2013) found a 30-μg/m^3^ increase in monitor PM_2.5_ was associated with a 19% (95% CI: 12, 23) increase in salbutamol dispensations in fire-affected populations of BC during the 2003 to 2010 fire seasons, using meta-regression from different LHAs. Henderson et al. (2011) reported that a 30-μg/m^3^ increase in monitor-observed PM_10_ was associated with a 16% increase in the odds of an asthma-specific physician visit in a cohort of 280,000 people during the 2003 wildfire season in BC. Although Henderson et al. (2011) used PM_10_ instead of PM_2.5_, the results are comparable because PM_2.5_ is the major fraction of PM_10_ from wildfire smoke (Moore et al. 2006; Wu et al. 2006). In that same study, a 30-μg/m^3^ increase in PM_10_ from wildfire smoke modeled by CALPUFF (in this case a retrospective model rather than an operational forecast) was associated with a 2% increase of odds of asthma-specific physician visits. Delfino et al. (2009) reported that a 30-μg/m^3^ increase in PM_2.5_ was associated with a 16% increase in asthma hospital admissions in Los Angeles, California.

Although BlueSky PM_2.5_ forecasts were consistently associated with health indicators and the effect estimates were comparable with Monitor PM_2.5_ measurements, the estimated effects were generally smaller and the CIs were narrower. An important contributor to this result was the large range of values in the BlueSky forecasts. When forecasts > 300 μg/m^3^ were truncated to 300 μg/m^3^ (approximately double the highest Monitor PM_2.5_ concentration), the point estimates were larger and the CIs were wider than those calculated with the original data (Table 2).

We also found a 5% increase in salbutamol dispensations associated with being covered by BlueSky or HMS plumes. For asthma-related physician visits, the increases were 6% and 9% for BlueSky and HMS Plumes, respectively. In the Henderson et al. (2011) cohort study, being covered by HMS plumes was associated with a 21% increase in the odds of an asthma-related physician visit. Rappold et al. (2011) used aerosol optical depth measured by satellites to identify dense smoke plumes in North Carolina. A 65% increase in asthma-specific emergency department visits was observed for smoke-affected counties when exposed days were compared with nonexposed days.

Larger point estimates of association were found for salbutamol dispensations in more smoky areas compared with less smoky areas, but this was not observed for asthma-related physician visits. This may suggest the increase in dispensations was more relevant to wildfire smoke exposures. Although only a few wildfire smoke studies (Caamano-Isorna et al. 2011; Elliott et al. 2013) have reported using pharmaceutical dispensation as an indicator of population health, our results further support the use of this indicator for wildfire smoke research and surveillance. However, although dispensations appear to be sensitive to smoke exposure, it is unclear whether the observed associations were driven by actual health impacts or by people filling prescriptions to prepare for smoke events. This might also be true for the physician visits.

## Conclusions

We found that agreement between BlueSky forecasts and observed data was reasonable compared with evaluations of other existing smoke forecasting systems. Better agreement was generally observed during intense fire periods. We also found significant associations between BlueSky forecasts and respiratory health outcomes, with risk estimates consistent with those calculated using the observed data and with those reported by other epidemiologic studies. These results suggest that BlueSky forecasts can provide useful information for public health decision making. Because the 2010 fire season was among the most extreme in the history of British Columbia, ongoing evaluation during typical fire seasons is needed to further validate the role of the BlueSky forecasts in public health protection.
